# Marginal Fit Evaluation of Zirconia Copings Based on Conventional and Digital Impressions Using a Tooth Section Microscopic Examination Technique

**DOI:** 10.3390/dj14020106

**Published:** 2026-02-12

**Authors:** Marc Schulze, Peter Ottl, Mareike Warkentin

**Affiliations:** 1 Department of Prosthodontics and Material Science, Faculty of Medicine, University of Rostock, Strempelstrasse 13, 18057 Rostock, Germany; peter.ottl@uni-rostock.de; 2 Working Group for Implant Materials, Faculty of Mechanical Engineering and Marine Technologies, University of Rostock, Friedrich-Barnewitz-Strasse 4, 18119 Rostock, Germany; mareike.warkentin@uni-rostock.de

**Keywords:** crown margin, marginal fit, impression technique, intraoral scan, tooth section

## Abstract

**Background/Objectives:** This study compared the marginal fit of CAD/CAM zirconia copings based on digital and conventional impression techniques by using a new method of tooth section examination. **Methods:** Twenty premolars were prepared for an all-ceramic full crown. Ten conventional impressions using the polyether Impregum and ten digital impressions using the cara TRIOS intraoral scanner were used to fabricate zirconia copings. Slices were prepared by cutting each tooth five times. The marginal fit was microscopically analyzed using seven defined distances at 20 measuring points per tooth. The statistical analyses were carried out by using the Mann–Whitney test and the Kruskal–Wallis test (α = 0.05). **Results:** The median values for the copings based on the conventional (c) and digital (d) impressions showed significant differences for the internal gap (c: 56 μm (IQR = 22); d: 30 μm (IQR = 13); *p* < 0.001), horizontal marginal discrepancy (c: 64 μm (IQR = 59); d: 84 μm (IQR = 41); *p* < 0.001), absolute marginal discrepancy (c: 81 μm (IQR = 40); d: 98 μm (IQR = 50); *p* < 0.001) and the overextension (c: 63 μm (IQR = 48); d: 89 μm (IQR = 50); *p* < 0.001). All copings based on digital impressions were overextended. Underextensions only occurred with the conventional impression method with a median value of 40 μm (IQR = 64). For the marginal gap (c: 29 μm (IQR = 27); d: 31 μm (IQR = 22); *p* = 0.477) and the vertical marginal discrepancy (c: 34 μm (IQR = 32); d: 35 μm (IQR = 40); *p* = 0.944) no significant differences could be found. **Conclusions:** Both impression methods are suitable for the fabrication of ceramic copings. The microscopic examination of tooth sections seems to be a suitable in vitro method for the evaluation of the marginal fit of ceramic copings.

## 1. Introduction

For conventional fabrication of dental crowns, it is necessary to transfer the clinical situation precisely to a physical cast. This transfer is performed by using different impression techniques. The established procedure is the usage of elastomeric impression materials to produce a plaster cast. But new technologies (additive and subtractive ones) and the corresponding dental materials, for example, used by the CAD/CAM technology, require a digital cast. This could be generated by scanning the physical plaster cast based on a conventional impression, or by direct digitization using an intraoral scanner. The marginal fit of a fabricated crown can be used as a criterion to compare the different impression methods and is important for the long-term success of dental crowns [1,2,3]. An insufficient marginal fit can lead to plaque accumulation and subsequently to secondary caries [4,5,6], periodontal diseases [4,6,7], and finally to the failure of the crown. Holmes et al. [8] defined seven relevant parameters of the marginal fit of crowns, which were applied in the present study. The marginal gap is defined as the vertical distance of the crown from the tooth in the marginal area [8]. It is one of the most important parameters for the quality of the crown, because it represents the area for the penetration of chemical and biological noxae. There is no agreement in the literature regarding the maximum size of an acceptable marginal gap [5,9]; it ranges from 20 μm [10] up to 120 μm [11]. In clinical studies, the measurements often surpass the required maximum values [9]. The absolute marginal discrepancy results from the combination of the marginal gap and the extension of the crown. This parameter has a great influence on the health of the periodontium [6].

In the literature different methods are used for examining the crown margin, but no standardized procedure is established: The simplest—but subjective—method is the visual inspection and examination of the crown margin using a dental probe [12]. The result depends on the individual tactility and experience of the examiner, the localization of the crown margin and the probe diameter [10,12]. Therefore, this technique is not reproducible and comparable. Furthermore, it is possible to examine the approximal area in vivo using a bitewing radiograph. But this method can lead to projection errors, and the situation can only be assessed from one angle of view. Optical coherence tomography [13] is a non-destructive [14], radiation-free method [15] that uses light reflection to visualize the marginal adaption of restorations [13], but the intraoral use is limited due to the size of the probe [15]. For the in vitro examination using Micro-CT, the crown does not have to be destroyed, and many measuring points are possible [16]. Otherwise this time-consuming and cost-intensive method features a resolution inferior to that of the microscope. Other examination methods include profilometry [4], laser videography [12], a virtual-fit method using different scanning technologies [17] and the analysis of direct digital photography of the crown margin [7]. A frequently used procedure is the replica method described by McLean and Fraunhofer [11]. It is applicable for in vivo and in vitro examinations as well. In the present study a new method of tooth sectioning in combination with a microscopic examination technique was used. Therefore, the aim of this study was two-fold: First, to compare the quality of the marginal fit of zirconia copings based on conventional and digital impressions using the parameters defined by Holmes et al. [8]—also with regard to the tooth side and the localization of the tooth slice—with a new examination method. Second, to explore whether this method is suitable for this purpose. The first null hypothesis is that the impression methods have no significant influence on the marginal fit quality of the fabricated restorations. The second null hypothesis is that the method of microscopic examination of tooth sections is suitable for evaluating the defined parameters.

## 2. Materials and Methods

**Figure 1:**
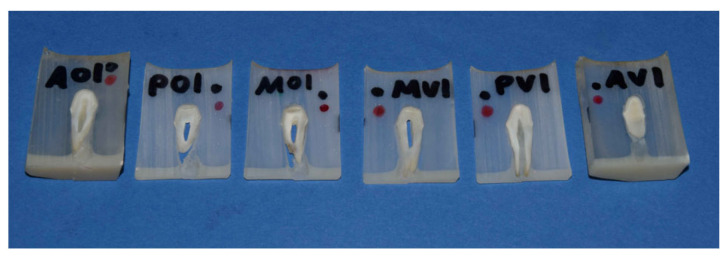
Tooth sections after cutting with precision diamond wire saw.

Because this study uses a new in vitro method for investigating the quality of the marginal fit, a pilot study was carried out in advance. The sample size was determined by using G*Power 3.1.9.7 (Heinrich-Heine-University Düsseldorf) (d = 0.8; α = 0.05). The required sample size is 35 per group. The use of 10 teeth for each impression method results in a sample size of 200 per group (see below). Twenty maxillary premolars—free of caries and restorations—were extracted due to periodontal or orthodontic indications. The alveoles in a study cast (KaVo Study Model Basic; KaVo, Biberach, Germany) were extended to fixate the extracted teeth samples using a polyether impression material (Impregum; 3 M, St. Paul, MN, USA). The preparation of the teeth was performed by the same person with a 0.5 mm supragingival chamfer using preparation diamonds (ISO 806 314 289525 + 012 and ISO 806 314 289514 + 012; Komet Dental/Gebr. Brasseler, Lemgo, Germany). The preparation angle was between 3 and 6 degrees. The occlusal reduction was 1.5 mm, and the circular axial substance removal was 1.2 mm. For 10 teeth conventional impressions were made with individualized impression trays using a polyether double mixing impression (Impregum; 3 M). Plaster casts have been fabricated using Type IV plaster (Fujirock EP; GC, Luzern, Switzerland) and a saw-cut system (giroform; Amann Girrbach, Mäder, Austria). These casts were digitized using a laboratory dental scanner (D 900 Dental Lab Scanner; 3Shape, Copenhagen, Denmark). The remaining 10 teeth were digitized directly with an intraoral scanner (cara TRIOS Cart intraoral scanner; Heraeus, Hanau, Germany). The zirconia copings (ceramill Zi; Amann Girrbach, Mäder, Austria) were computer-aid designed (ceramill Mind; Amann Girrbach, Mäder, Austria) according to manufacturer’s instructions (0.5 mm minimum frame thickness, 0.2 mm crown margin thickness (horizontal), 60° angle, 0 mm height (vertical)) with a cement space setting of 0.017 mm. This was followed by the fabrication of the copings by using a 5-axis milling machine (ceramill Motion 2; Amann Girrbach, Mäder, Austria) in a dental laboratory. First, the copings have been checked visually and tactilely both on plaster dies as well as on the prepared tooth by using a stereo light microscope (Stemi DV 4; Carl Zeiss, Oberkochen, Germany) and a dental probe (Silver Line Explorer Probe No. 5 123-010; Hu-Friedy, Chicago, IL, USA). The cementation was performed with a self-adhesive resin cement (RelyX Unicem Automix; 3 M, St. Paul, MN, USA), and the copings were fixed by finger pressure until the end of the setting time. The twenty teeth with the cemented copings were embedded in cold-curing epoxy resin (EpoThin 2 Epoxy Resin; Buehler, Lake Bluff, IL, USA) and then cut five times using a water-cooled diamond wire saw (Precision Diamond Wire Saw 3241; Well Diamond Wire Saws SA, Mannheim, Germany), which enabled parallel cutting by a micrometer screw. It was not possible to make more cuts because the slices would have been too thin, and the parts such as tooth, ceramic and epoxy resin would delaminate. The first cut was made perpendicular and centrally through the tooth. The cut direction was mesial to distal. Two additional slices were cut from each of the resulting two parts using a micrometer screw at the sawing device to enable precisely parallel cuts. The average thickness of the slices was 86 μm. As a result, each tooth was separated into six slices (Figure 1). Throughout the entire process of preparing and evaluating the tooth sections—from embedding and cutting to microscopic examination—markings were used on the samples to ensure accuracy in terms of tooth side and orientation.

**Figure 2:**
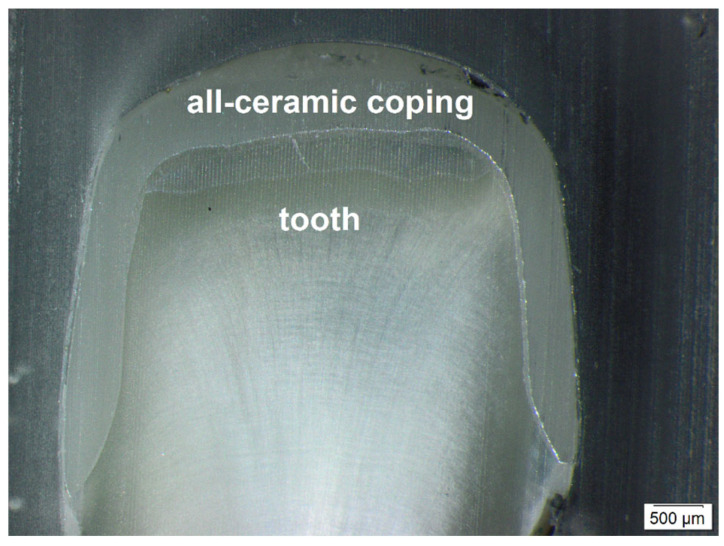
Light microscopic image of the coronal tooth part.

**Figure 3:**
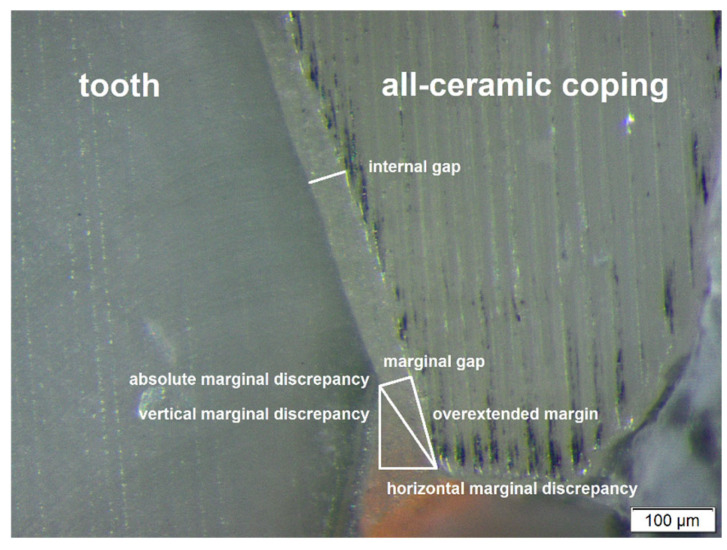
Example of microscopic measured marginal gap geometry according to Holmes et al. [8].

The two central and two peripheral slices had a front and a back side. In contrast to these inner segments, the two outer segments had only one side, which was examined. This resulted in ten surfaces, each with a mesial and distal side. Finally, 20 measuring points per tooth were collected. The examination was performed using a binocular stereo zoom microscope with 126× magnification (Olympus SZX 10; Olympus, Tokyo, Japan) and the related software (cellSens Life Science Imaging; Olympus, Tokyo, Japan) (Figure 2 and Figure 3). In summary, 200 measuring points for each impression method were assessed to evaluate the seven parameters defined by Holmes et al. [8] (Figure 3) at each of these points.

The statistical data analysis was performed using IBM SPSS Statistics for Windows, v27.0 (IBM). The Kolmogorov–Smirnov test was performed to determine the normality of the distribution of the data. The data were not normally distributed. Bias-corrected and accelerated 95% bootstrap confidence intervals were determined. The Mann–Whitney test was used for the comparison of the impression methods and for the comparison of the two tooth sides. The Kruskal–Wallis test was performed to investigate the influence of the different localizations of the tooth slices. The correction for multiple comparisons was performed by using the Bonferroni correction. Pearson’s correlation was used for the evaluation of the effect size. The significance level was α = 0.05.

## 3. Results

**Figure 4:**
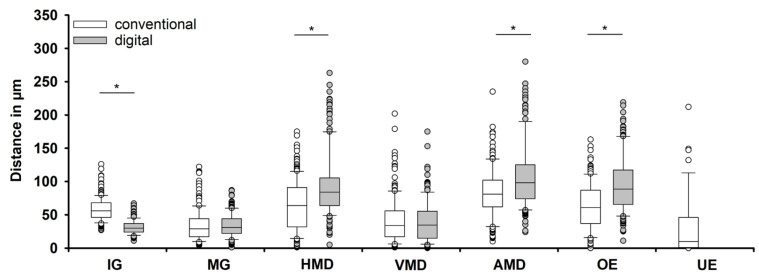
Comparison of seven marginal fit parameters [8] based on conventional and digital impressions: (IG) internal gap, (MG) marginal gap, (HMD) horizontal marginal discrepancy, (VMD) vertical marginal discrepancy, (AMD) absolute marginal discrepancy, (OE) overextension, and (UE) underextension. Statistical significance is indicated by asterisks.

The seven marginal gap parameters according to Holmes et al. [8] were assessed and compared for both impression methods (Figure 4) (Table 1 and Table 2). The median value for the internal gap was significantly (*p* < 0.001; r = 0.74) larger using the conventional impression method (56 μm (IQR = 22)) in comparison with the digital impressions (30 μm (IQR = 13)). For the horizontal marginal discrepancy, a significantly (*p* < 0.001; r = 0.31) smaller median value was found for the conventional impressions (64 μm (IQR = 59)) compared to the intraoral scans (84 μm (IQR = 41)). Similar results were found for the median value for the absolute marginal discrepancy, which was significantly (*p* < 0.001; r = 0.24) smaller for the polyether impression (81 μm (IQR = 40)) compared to the digital technique (98 μm (IQR = 50)). When comparing the overextensions, the median value of the digital impression (89 μm (IQR = 51)) was significantly (*p* < 0.001; r = 0.36) larger than the median value of the conventional elastomer impression (63 μm (IQR = 48)). All copings based on digital impressions were overextended. Underextensions only occurred with the conventional impression method. The median value for these parameters was 40 μm (IQR = 64). The median values for the marginal gap (conventional: 29 μm (IQR = 27); digital: 31 μm (IQR = 22); *p* = 0.477; r = 0.04) and the vertical marginal discrepancy (conventional: 34 μm (IQR = 32); digital: 35 μm (IQR = 40); *p* = 0.944; r = 0.00) showed no significant differences.

Further investigations were carried out focusing on two important parameters from a clinical point of view. The marginal gap and the absolute marginal discrepancy: the first parameter represents the real gap at the crown margin, and it is not influenced by the extension. The absolute marginal discrepancy results from the horizontal and vertical marginal discrepancies and includes the extension. The marginal fit of each tooth side was evaluated. For this purpose, the measurements of the mesial side were compared with those of the distal side (Table 3).

The median values for the marginal gap did not differ significantly from each other, neither for the mesial (*p* = 1; r = 0.01) nor for the distal (*p* = 0.769; r = 0.07) tooth side. Within the group of conventional (*p* = 0.529; r = 0.14) as well as digital (*p* = 0.315; r = 0.23) impressions, there were no significant differences regarding the tooth side to be found. The examination of the absolute marginal discrepancy showed significant differences between the median values mesial (*p* < 0.05; r = 0.47) as well as distal (*p* < 0.01; r = 0.07). Within the group of conventional (*p* = 0.529; r = 0.14) and digital (*p* = 0.739; r = 0.08) impressions, there were no significant differences between the two sides of the teeth.

Finally, it was investigated whether the localization of the slices affects the dimension of the gap parameters (Table 4 and Table 5).

As previously described, the teeth were cut centrally, and two additional slices were cut from each half. This resulted in one central and one peripheral slice per tooth half and the remaining outer part. The median values of the marginal gap did not show any significant differences regarding the localization of the slices (*p* > 0.05) for the conventional and the digital impressions. The examination of the median values of the absolute marginal discrepancy showed no significant differences for the slices based on conventional impressions. For digital impressions, the differences between the central and peripheral slices were significant (*p* < 0.05). Between the central and outer (*p* = 0.068) as well as the peripheral and outer (*p* = 0.935) slices no significant differences were found.

## 4. Discussion

Concerning the measurement of the marginal fit, several studies refer to the work of Holmes et al. [8]. But the exact specifications, which of the seven parameters were investigated, are often missing. Instead of this, a general term is mentioned. This complicates the comparability of studies regarding the marginal fit.

The marginal gap determines the extent of cement leaching from the cement gap and the penetration of microorganisms [3,10]. It was possible to fabricate copings with approximately similar values for the marginal gap using the conventional and digital impression methods. Numerous studies [2,3,7,14,18] quoted a result of 120 μm—mentioned by McLean and Fraunhofer [11]—as clinically acceptable for the marginal gap. The present study was able to show that it is possible to fabricate ceramic copings with a marginal gap below 120 μm with conventional as well as digital impression techniques.

Another important parameter for the evaluation of the marginal fit is the extension of the fabricated crown. Overextension can lead to plaque accumulation and periodontal disease; underextension can lead to secondary caries due to the exposed tooth structure. This study showed that copings based on digital impressions without post-processing by a dental technician on a physical cast had significantly larger overextensions than copings based on conventional impressions and post-processing. Underextensions were only present in the copings after conventional impression. These results can be explained by the workflow used in this study. The ceramic copings based on conventional impressions were all checked and manually corrected by a dental technician. Because of the lack of a physical cast and the resulting impossibility of correction all copings based on digital impressions showed overextensions.

The absolute marginal discrepancy is a combination of the marginal gap and the extension. Due to the overextension of all copings based on digital impression, the absolute marginal discrepancy had a larger median value (98 μm) compared to the conventional method (81 μm). Other in vitro studies, as well as meta-analyses found marginal discrepancies between 48 μm [1] and 138.17 μm [5] for elastomeric impressions. For restorations based on intraoral scans, marginal discrepancies of 48 μm [19] up to 151.68 μm [5] were found.

In addition to the peripheral located parameters (marginal gap and absolute marginal discrepancy), the internal gap also has an important influence on the success of a dental restoration. The comparison of the effect size showed that the impression method has a major influence on the internal gap. This is important because a large internal gap increases the thickness of the cement layer and can have a negative effect on the mechanical stability of a zirconia restoration [20]. Nevertheless, no clinical acceptable value for this parameter is established [21].

The confidence intervals for the parameters of the marginal fit were quite narrow with a tendency toward low values. This can be considered positive, as small values for the dimensions of the parameters of the marginal fit are clinically important and desirable. An optimal marginal fit increases the success rate of fixed dental restorations, as it reduces plaque accumulation and the penetration of microorganisms, thereby reducing the risk of gingivitis, periodontitis, and secondary caries.

The idea of examining the influence of the tooth side on the parameters of the marginal fit arose due to the different accessibility of these areas in clinical treatment. Between the mesial and distal sides of the teeth, there was no significant difference concerning the marginal fit of the fabricated ceramic copings. This can be explained by the fact that this study was performed in vitro. Thus, ideal conditions for tooth preparation and impression making were realizable. In contrast to the clinical treatment of a patient, the absence of patient movement, saliva and blood, as well as good accessibility, illumination and better visibility were provided.

Considering the results regarding the localization of the prepared tooth slices, the marginal gap showed a slight increase in the median values from the central slices to the external ones. This does not apply to the absolute marginal discrepancy, because it is more influenced by the extension and less by the gap geometry and its distortion by the sectional plane.

The investigation of crown copings for the evaluation of the marginal fit is an established procedure [20,22,23,24,25]. For this study Zirconia copings were fabricated. Due to the hardness of this material, a monolithic restoration would have made it much more difficult or even impossible to create the tooth sections.

In fixed prosthodontics a perfect marginal fit prevents secondary caries and periodontal diseases and thus, contributes to the long-term success of dental crowns [1,2,3]. As mentioned above, there is no ISO or DIN-regulated procedure for the investigation of the marginal fit of dental crowns. Some studies use the method of making tooth sections, but only a few [22,26] use real teeth. Furthermore, in these studies, the sample was either cut only once [20,22,23,27,28] or twice (cross-sectioning) [24,25,26]. The authors are unaware of investigations using the presented method of preparing slices of embedded real teeth with crown copings for the examination of the marginal fit. A method like the frequently used replica method [11] examines the quality of the marginal fit indirectly by using an elastomer impression of the inside of the restoration. The method used in this study enables a direct visualization and measurement of the involved structures and necessary parameters according to Holmes et al. [8]. This complex procedure of tooth sectioning and the following microscopic examination permits a high precision in the determination of the measurement points as well as the measurement itself [29,30]. But it leads to the destruction of the samples, so it could only be used in vitro. As an alternative to this, Micro-CT is a non-destructive in vitro method. It is an accurate, reliable, and repeatable method [16], but it is associated with high costs [21,31] and relatively low resolution [21] compared to light microscopic examination. Investigations of the marginal fit using the Nano-CT may provide better resolutions in the future [18].

There is no reliable information on how many samples are required for the evaluation of the marginal gap [7]; many studies use n ≤ 10 specimens for each group [7,14,18,21,23,28,30,31]. We have consulted the literature and performed a pilot study as well as a G*Power Analysis to determine the sample size for this study. Moreover, there is no agreement in the literature regarding the number of measurements required for the evaluation of the marginal fit [3,32]. Many measurements are advantageous for the statistical data analysis. Measurements of four sites, for example, vestibular, oral, mesial and distal, are not adequate [7]. One study [30] used perpendicular sections along the tooth axis, which resulted in eight measurement points. Another study [7]—using direct microscopic investigation of the crown margin—described 50 measuring points for each crown. The method of five parallel longitudinal sections, as used in the present study, results in 20 measuring points per tooth. Using 10 teeth for each impression method, 200 measuring points for the investigation of the polyether impression as well as the digital impression were achieved. It was not possible to generate more measuring points by preparing more sections because thinner slices would be fragile and the components, tooth, ceramic and epoxy resin would separate or fracture during the sawing process.

There are some limitations to this study. First of all, this study was conducted in vitro. On the one hand, this type of research allows dental crowns to be evaluated under optimal conditions without patient-related factors such as salivation and bleeding, in order to obtain a valid assessment of the quality of the marginal fit of crowns. On the other hand, in vitro studies do not reflect clinical conditions such as the complex process of patient treatment. Other limitations include the use of only one intraoral scanner and the different processing of the copings due to the lack of a physical model in intraoral scanning technology. The fact that only the presented method of microscopic examination of tooth sections for the evaluation of the marginal fit was used should be considered as a limitation of this study. Moreover, only a limited number of sections can be generated by preparing these tooth sections. Further studies comparing the described method with other examination techniques would be useful to verify the reliability of the method presented here.

## 5. Conclusions

Conventional double mix impressions using polyether as well as the usage of digital impressions using an intraoral scanner allowed the fabrication of ceramic copings with median values below the frequently required 120 μm. The preparation of tooth slices and their examination using a light microscope seems to be a suitable method for analyzing the quality of the marginal fit; the parameters defined by Holmes et al. [8] have proven their worth here.

## Figures and Tables

**Table 1:** Results of marginal fit parameters [8] based on conventional impressions in µm.

Parameter of Marginal Fit [8]	Minimum	BCa95% LCL	Mean	BCa95% UCL	Maximum
Internal gap	27	55	58	60	126
Marginal gap	0	31	35	38	122
Horizontal marginal discrepancy	1	59	64	70	175
Vertical marginal discrepancy	1	37	42	46	202
Absolute marginal discrepancy	10	78	84	89	235
Overextension	0	60	65	70	163
Underextension	0	39	56	74	212

**Table 2:** Results of marginal fit parameters [8] based on digital impressions in µm.

Parameter of Marginal Fit [8]	Minimum	BCa95% LCL	Mean	BCa95% UCL	Maximum
Internal gap	11	30	31	33	67
Marginal gap	1	31	34	36	87
Horizontal marginal discrepancy	5	88	95	103	263
Vertical marginal discrepancy	0	36	40	46	175
Absolute marginal discrepancy	24	101	108	116	280
Overextension	11	89	96	102	219

**Table 3:** Results of two marginal fit parameters [8] based on conventional and digital impressions depending on tooth side in µm.

		Conventional Impression			Digital Impression		
Parameter of Marginal Fit [8]	Tooth- Side	25%	Median	75%	25%	Median	75%
Marginal gap	mesial	17	30	47	24	32	47
	distal	17	27	43	21	29	39
Absolute marginal discrepancy	mesial	64	82	108	75	99	133
	distal	61	80	98	76	96	118

**Table 4:** Results of two marginal fit parameters [8] based on conventional impression depending on localization of tooth slice in µm.

	Marginal Gap [8]			Absolute Marginal Discrepancy [8]		
Localization of the Slice	25%	Median	75%	25%	Median	75%
Central	17	30	43	62	78	98
Peripheral	16	29	44	62	85	104
Outer	19	35	53	63	81	109

**Table 5:** Results of two marginal fit parameters [8] based on digital impression depending on localization of tooth slice in µm.

	Marginal Gap [8]			Absolute Marginal Discrepancy [8]		
Localization of the Slice	25%	Median	75%	25%	Median	75%
Central	23	32	43	85	104	146
Peripheral	19	29	39	73	94	104
Outer	26	35	47	67	93	132

## Data Availability

The original data presented in the study are openly available in RosDok at https://doi.org/10.18453/rosdok_id00004895.

## Abbreviations

The following abbreviations are used in this manuscript:

AMD	absolute marginal discrepancy
BCa	bias-corrected and accelerated
HMD	horizontal marginal discrepancy
IG	internal gap
LCL	lower confidence limit
MG	marginal gap
OE	overextension
UCL	upper confidence limit
UE	underextension
VMD	vertical marginal discrepancy
